# How Can We Prove the Causality of Interbrain Synchronization?

**DOI:** 10.3389/fnhum.2021.651949

**Published:** 2021-02-25

**Authors:** Hila Z. Gvirts Provolovski, Rotem Perlmutter

**Affiliations:** The Department of Behavioral Sciences and Psychology, Ariel University, Ariel, Israel

**Keywords:** interbrain synchronization, neurofeedback, social alignment, hyperscanning, multibrain stimulation

Recently, Novembre and Iannetti (2020) highlighted a fundamental question concerning interbrain synchrony (IBS; the observed synchronization between two or more brains during social interactions). The concern is whether IBS serves as a neural mechanism that causally facilitates social interactions, or whether it is simply an epiphenomenon, arising from the fact that participants are exposed to the same stimuli, thus eliciting similar brain activations in each brain. This is an important topic, in which we have quite some interest as well.

Previously (Gvirts and Perlmutter, 2019), we aimed to characterize the unique phenomenon of IBS, stressing that it is greater than the sum of the mere activations of similar neural regions within each individual brain, as it facilitates more attunement and greater allocation of attention to the interaction. This is done in order to increase the interaction's potential gains. We find great support for this notion in different hyperscanning studies, showing that even when participants were exposed to the same stimuli, it was only when their interaction was perceived as “significant” and created a sense of shared intentionality that they exhibited marked IBS. Fishburn et al. (2018) defined shared intentionality as the shared goals, which direct how participants coordinate their efforts in collaborative interactions. They managed to show its importance in a hyperscanning study, by observing an increase in IBS between the prefrontal cortices (PFC) of participants who were engaged in actions toward a mutually held goal, compared to identical tasks undertaken individually. In another study (Lu and Hao, 2019), in which one of the three group participants was a confederate pretending to participate in the study, a greater IBS was found only between the dorsolateral PFCs of the real participants. Finally, Liu et al. (2016) manipulated different levels of interaction and intentionality within a Jenga game, with participants (1) playing the game alone, parallel with one another; (2) playing the game cooperatively; (3) playing the game in an obstructive manner. Importantly, IBS was not present during the parallel playing condition. Moreover, while both interactive conditions in the study led to IBS in the middle frontal gyrus (MFG) and superior frontal gyrus (SFG), an additional IBS appeared between the dorsomedial PFCs only in the cooperative condition.

Taken together, these findings offset the criticism that greater IBS merely reflects similar task demands across the participants (Lu and Hao, 2019). Moreover, they highlight the importance of shared intentionality, and its great effect upon IBS.

Hence, we are not merely discussing the incidental common activations between two (or more) brains. This joint activation marks something uniquely different, which we believe (with support from hyperscanning studies) facilitates the achievement of social goals (Gvirts and Perlmutter, 2019).

Novembre and Iannetti (2020) suggest that hyperscanning studies, such as those mentioned above, are not enough to resolve the question of causality, as they can only imply correlation. This is a valid point. To resolve it, they propose stimulating similar regions in different individuals' brains, and then observing whether this elicited better outcomes in a consequent social interaction. They also suggest that hyperscanning can be used to note whether IBS occurs following such stimulation. This approach does seem to address the potential criticism that IBS arises from similar external stimuli or task demands. However, it does not address our notion that IBS is more than a co-occurrence of congruent neural regions. First and foremost, their proposed methodology excludes a crucial element of IBS- an engagement in social interaction. Second, as we stated above, previous hyperscanning studies strongly point to the fact that there has to be some shared intentionality mediating/leading the process of synchronization. It is therefore reasonable to assume that simply stimulating congruent neural regions within each individual brain, without an engagement in *interaction*, might be null.

We conclude that the difference between simultaneous stimulations of congruent neural regions and actual IBS is that IBS, as it occurs during our everyday social interactions, necessitates participants to interact with one another.

Hence, we suggest that in order to investigate the causality question of whether IBS is a mechanism or an epiphenomenon of social interactions, we must do so in the context of social interactions. We propose to do this by directly and exogenously manipulating IBS through brain-to-brain neurofeedback modulations in naturalistic settings. That is to say, while participants are engaged in social interaction. If this results in greater attunement between the participants and in greater social achievements from the interaction, this can be seen as strong evidence toward causality. The difference between our suggestion and that of Novembre and Iannetti (2020) is in the actual activation of synchronization, not the mere activation of similar regions, temporally controlled, in the hopes of achieving synchronization.

Previous studies (e.g., Duan et al., 2013; Zhang and Zhao, 2018) showed the feasibility of brain-to-brain neurofeedback methodologies in training IBS between participants. Excitingly, such a training system was recently found to increase IBS and its prosocial implications over time in pigeons (Yang et al., 2020). In these studies, training was achieved via a separate game or a reward system, prior to the investigated interaction. We believe it is possible to utilize these methodologies under more ecological settings, by delivering feedback to participants on their levels of IBS directly within the explored social interaction (e.g., card playing and talking). If participants are then able to increase their IBS, and this results in greater prosocial outcomes or greater motor and emotional alignment (Shamay-Tsoory et al., 2019), this can serve as very strong evidence toward causality (see Figure 1). That is to say, that IBS is the facilitator, not the epiphenomenon, of social interactions.

**Figure 1:**
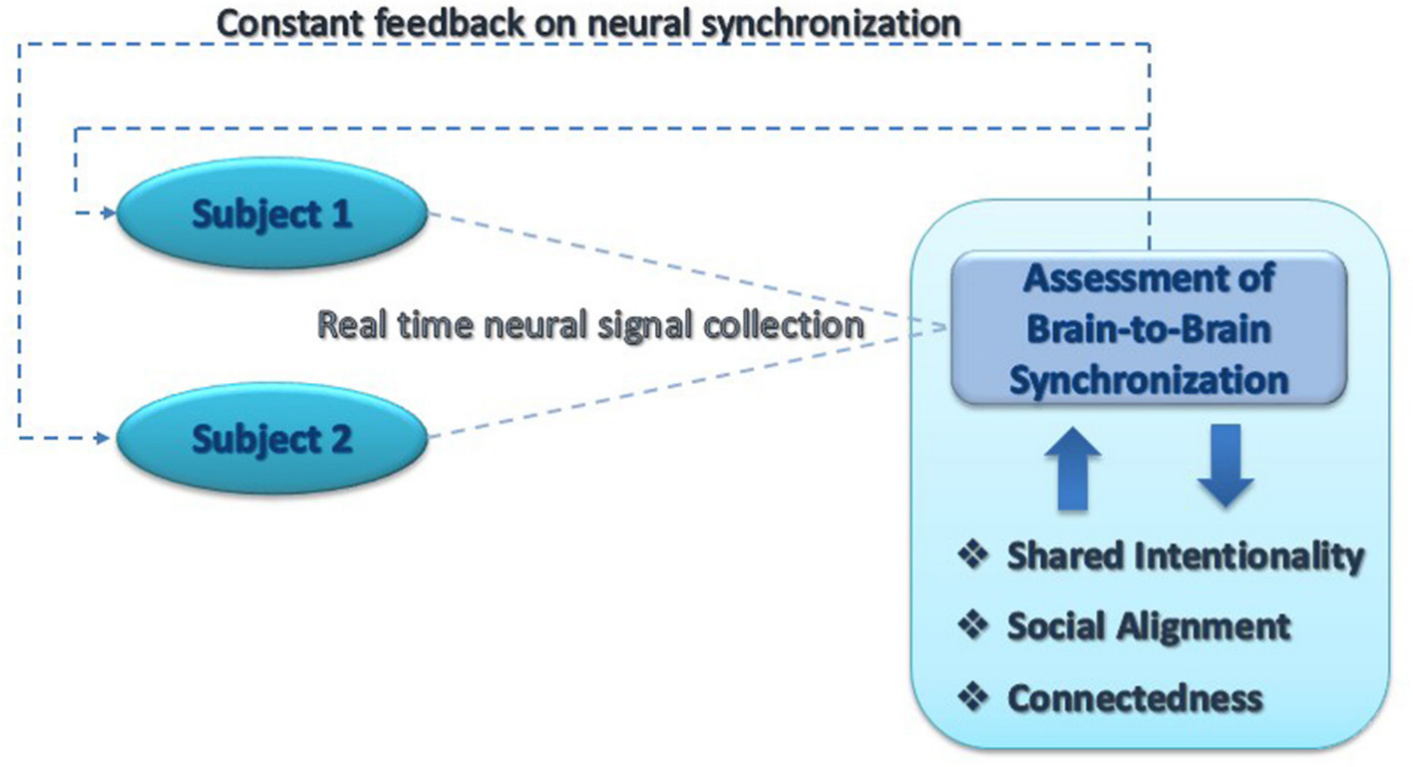
A brain-to-brain neurofeedback system under ecological settings. A schematic proposal of utilizing online hyperscanning during naturalistic interactions to measure the level of IBS (interbrain synchrony) between the participants. Participants then receive ongoing feedback regarding their IBS levels, and are encouraged, and thus trained, to increase them. The interaction itself is viewed and its social and individual aspects are measured, in order to determine causality, i.e., whether increasing IBS consequently increases different social measures, such as alignment and cooperation, shared intentionality, and social connectedness.

Such studies might hold more than theoretical implications. In fact, if IBS is indeed the mechanism by which we can induce greater levels of shared intentionality and prosociality, naturalistic IBS training sessions can also be implemented as a treatment method for different disorders associated with social deficits, such as autism.

## Author Contributions

All authors listed have made a substantial, direct and intellectual contribution to the work, and approved it for publication.

## Conflict of Interest

The authors declare that the research was conducted in the absence of any commercial or financial relationships that could be construed as a potential conflict of interest.
